# Olivine Weathering in Soil, and Its Effects on Growth and Nutrient Uptake in Ryegrass (*Lolium perenne* L.): A Pot Experiment

**DOI:** 10.1371/journal.pone.0042098

**Published:** 2012-08-09

**Authors:** Hein F. M. ten Berge, Hugo G. van der Meer, Johan W. Steenhuizen, Paul W. Goedhart, Pol Knops, Jan Verhagen

**Affiliations:** 1 WUR Plant Research International, Wageningen, The Netherlands; 2 WUR Biometris, Wageningen, The Netherlands; 3 Innovation Concepts B.V., Gorinchem, The Netherlands; Utrecht University, The Netherlands

## Abstract

Mineral carbonation of basic silicate minerals regulates atmospheric CO_2_ on geological time scales by locking up carbon. Mining and spreading onto the earth's surface of fast-weathering silicates, such as olivine, has been proposed to speed up this natural CO_2_ sequestration (‘enhanced weathering’). While agriculture may offer an existing infrastructure, weathering rate and impacts on soil and plant are largely unknown. Our objectives were to assess weathering of olivine in soil, and its effects on plant growth and nutrient uptake. In a pot experiment with perennial ryegrass (*Lolium perenne* L.), weathering during 32 weeks was inferred from bioavailability of magnesium (Mg) in soil and plant. Olivine doses were equivalent to 1630 (OLIV1), 8150, 40700 and 204000 (OLIV4) kg ha^−1^. Alternatively, the soluble Mg salt kieserite was applied for reference. Olivine increased plant growth (+15.6%) and plant K concentration (+16.5%) in OLIV4. At all doses, olivine increased bioavailability of Mg and Ni in soil, as well as uptake of Mg, Si and Ni in plants. Olivine suppressed Ca uptake. Weathering estimated from a Mg balance was equivalent to 240 kg ha^−1^ (14.8% of dose, OLIV1) to 2240 kg ha^−1^ (1.1%, OLIV4). This corresponds to gross CO_2_ sequestration of 290 to 2690 kg ha^−1^ (29 10^3^ to 269 10^3^ kg km^−2^.) Alternatively, weathering estimated from similarity with kieserite treatments ranged from 13% to 58% for OLIV1. The Olsen model for olivine carbonation predicted 4.0% to 9.0% weathering for our case, independent of olivine dose. Our % values observed at high doses were smaller than this, suggesting negative feedbacks in soil. Yet, weathering appears fast enough to support the ‘enhanced weathering’ concept. In agriculture, olivine doses must remain within limits to avoid imbalances in plant nutrition, notably at low Ca availability; and to avoid Ni accumulation in soil and crop.

## Introduction

The United Nations Framework Convention on Climate Change aims at stabilizing greenhouse gas concentrations in the atmosphere at a level that would prevent dangerous anthropogenic interference with the climate system [1]. To achieve this goal it is imperative to move towards a low-carbon economy. All sectors of economy, including agriculture and forestry, will have to play a role in facilitating this transition.

Of the two major natural pathways that regulate atmospheric CO_2_ by carbon sequestration, the weathering of minerals (‘mineral carbonation’) has received less attention than photosynthesis and the organic matter cycle associated with it. Indeed, increased storage of carbon in biomass and soil organic matter can help reduce atmospheric CO_2_ concentration. Soils in their natural state retain substantial amounts of organic carbon for longer time periods [2], [3], but building-up soil organic carbon stocks is difficult, and is limited by saturation levels that depend on local conditions such as soil type, drainage, temperature and rainfall [3]. On a geological time scale, the weathering of basic silicate rocks and subsequent precipitation of Ca- and Mg-carbonates is the main process controlling CO_2−_concentration in the atmosphere. Along with plate tectonics - folding carbonate deposits back into the mantle – it constitutes the earth's thermostat [4], [5]. Utilizing this geochemical cycle to reduce atmospheric CO_2_ concentration, then, seems a logical option to counteract anthropogenic emissions. This was proposed by [5], who introduced the term ‘enhanced weathering’ for the large scale mining, grinding and spreading of silicate rocks such as olivine (Mg_2_SiO_4_), that can react with CO_2_ relatively fast. Olivine and its metamorphic counterpart serpentine are available in large quantities in the earth's mantle, and are accessible for mining at many locations on various continents. Within Europe, huge reserves are accessible in Norway, Sweden, Spain, Italy, Austria, Greece and Turkey [5].

Although the efficiency and applicability of this option is debated, the process of weathering itself and the consequent reduction of CO_2_ in the atmosphere are not [6], [7]. Exposed to water and CO_2_, olivine reacts with CO_2_ to produce a magnesium bicarbonate solution:
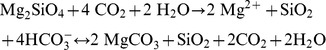
(1)


While part of the bicarbonate anions can be neutralised by soil acids sending CO_2_ back into the atmosphere, the remainder may precipitate *in situ* or may be leached from terrestrial systems and ultimately precipitate in the oceans, thus forming limestones and dolomites that together hold some 80% of our planet's carbon stock [4].

With almost five billion ha, agriculture uses about thirty six percent of the world's land area [8], and could provide an existing infrastructure for the implementation of the ‘enhanced weathering’ idea [5], [7]. Nutrient application is common practice on large tracts of land, and the use of rock minerals as fertiliser is not new either [9]. While olivine weathering under laboratory conditions is well documented [10], no experimental data are available on enhanced weathering in soil under cropped conditions.

We hypothesized that weathering of olivine in soil can contribute substantially to CO_2_ sequestration without negative impact on plant growth. Our first objective was to determine the rate of weathering of olivine powder in soil. The second objective was to test how olivine affects plant growth and nutrient uptake. For the semi-natural conditions of a pot experiment, these objectives were achieved by measuring changes in magnesium (Mg) content in soil and crop, and using these to infer olivine weathering. We also studied effects of olivine on plant growth and uptake of selected elements, and on bioavailability of Mg, silicon (Si) and nickel (Ni), the latter being one of the trace metals substituting for Mg in the olivine crystal.

## Materials and Methods

### Pot experiment

A pot experiment was conducted during 32 weeks at Wageningen, the Netherlands. The seven treatments (Table 1) in four replicates (blocks) included five doses of olivine powder (including zero). Olivine doses increased fivefold each next level. Two levels of kieserite, a highly soluble Mg sulphate fertiliser, were included. We used kieserite as a reference expected to provide its full Mg content (16.2% on mass basis) as bioavailable Mg right from the start of the experiment. Our forsterite-dominated olivine product from ‘North Cape Olivine Sand’ (Sibelco Nordic Ltd.) contained 23.4 mass % Mg, and 4.0 mass % Fe (this corresponds to a molar Fe∶Mg ratio of 1∶13.4). See also Table S1 for chemical composition. The finely-ground olivine product consisted of 7 mass-% of particles <2 µm, 66% between 2 and 50 µm, and 27% between 50 and 200 µm. The full particle size distribution curve is given in Figure S1. The olivine powder was further characterised by a BET (Brunauer, Emmett and Teller) specific surface area of 4.8 m^2^ g^−1^, determined by N_2_ adsorption [11] at 77 K on a Micromeritics TriStar 3000 analyzer. (Prior to adsorption measurement, the olivine sample was degassed in vacuum for 16 hours at 120°C and 350°C, respectively. The two temperatures yielded the same BET areas.)

**Table 1:** Treatments in pot experiment, Wageningen, 2009–2010.

Treatment name	Product	Product dose (g/pot)	Magnesium contained in product dose (g/pot)
Control	None		0
KIES1	Kieserite	1.5	0.24
KIES2	Kieserite	3.0	0.49
OLIV1	Olivine	8.0	1.88
OLIV2	Olivine	40.0	9.4
OLIV3	Olivine	200	47.0
OLIV4	Olivine	1000	235.0

The olivine powder was mixed with sandy soil collected from a field under arable cultivation at Droevendaal farm near Wageningen. The soil was selected for its relatively low bioavailability (among Dutch arable soils) of Mg (44.4 mg kg^−1^) and K (41.7 mg kg^−1^). Further chemical characteristics of the soil are given in Table S2.

PVC pots of 10 litre, with top diameter of 250 mm, were filled with the soil-olivine mixture. Pots contained 11.0 kg dry soil, except at the highest olivine rate where 9.5 kg soil was used. We used perennial ryegrass (*Lolium perenne*, cv. Barata) as the test crop, for its capacity to absorb large amounts of nutrients over a long growth period, and for the elasticity of nutrient contents in its biomass. The grass was sown on 20 August 2009, at 0.3 g seed per pot. At the start, all pots were supplied with adequate initial amounts of nitrogen (N), phosphorus (P), and potassium (K), equivalent with rates of 80 kg N ha^−1^, 19.6 kg P ha^−1^, and 80 kg K ha^−1^. Fertilisers were finely ground and mixed through the upper half of soil in the pots. To sustain high biomass production, extra N, P and K were supplied after each harvest as finely ground fertilisers on top of the remaining grass stubble, and flushed into the soil with irrigation water. See Table S3 for a full account on fertiliser management. To avoid the possibility of nutrient losses via downward percolation, irrigation water was supplied daily onto a tray below each pot, for a predominantly upward flow. Once every 14 days, 400 ml water was supplied on top to prevent accumulation of solutes near the surface.

Pots were kept outdoors under transparent rain shelter, until 3 November. They were then transferred to a greenhouse to sustain crop growth and soil processes during winter and early spring. The temperature regime for both periods is given in Figures S2 and S3. Always leaving the grass stubble for regrowth, we harvested aboveground plant biomass on 28 September, 2 November, 3 December, 11 January, 22 February, and 31 March. Fresh biomass yield was measured, and samples were dried at 70°C to assess dry matter yield. The experiment ended on 7 April, 2010. The soil from each pot was then thoroughly mixed and sampled for chemical analysis.

Plant samples were analysed separately (per harvest, per pot) for N, P, K, Ca, Mg, S, Ni, Fe, and Si. Soil was analysed for total and bioavailable contents of these elements, at the end of the experiment. Methods of soil destruction, element extraction (bioavailability), and elements analysis in soil and crop are given in Table S4. Soil water samples were collected only occasionally, and from selected treatments. This was done by suction using a 100 mm long synthetic microporous tube, embedded horizontally halfway between top and bottom in the centre of each pot. Water samples were analysed for pH and total alkalinity, for Mg and Ni (two occasions), and Si (once).

### Estimation of the fraction of olivine weathered

We estimated olivine weathering in our experiment by two methods. Method 1 is based on the Mg balance: the amount of olivine weathered corresponds to the amount of bioavailable Mg accumulated in soil (*Mg*_bio,soil_, g pot^−1^) and plant biomass (*Mg*_plant_, g pot^−1^), in excess of that in the Control. The mass fraction of olivine weathered (*F*_weath,Meth1_) relative to olivine applied, is then written as:
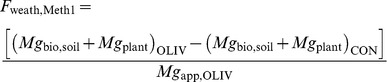
(2)where the subscripts of groups in brackets refer to treatments with olivine (OLIV) and control (CON). The symbol *Mg*_app_,_OLIV_ refers to the amount of Mg applied in the form of olivine (g pot^−1^).

Alternatively (Method 2), the kieserite treatments were used as references. This enables to express an olivine dose as an equivalent kieserite dose, that is, a dose that has the same impact on a particular response variable, *X*. Thus, we can calculate by interpolation (between Control and KIES1; or between KIES1 and KIES2) or by extrapolation (beyond KIES2) how much Mg in kieserite form was needed to achieve the effect on *X* found in OLIV1. Presuming full dissolution of kieserite, then, the same amount of Mg must have been dissolved from olivine. For example, if the response variable *X* in treatment OLIV1 is between the *X* values found in treatments KIES1 and KIES2, respectively, then the mass fraction of olivine weathered (*F*_weath,Meth2_) in OLIV1 is calculated as:
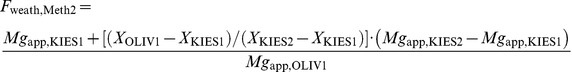
(3)where subscripts again refer to treatments, and *Mg*_app_ are amounts (g pot^−1^) of magnesium applied in the respective treatments (KIES1, KIES2, OLIV1). In contrast to Method 1, this Method 2 does not rely on a complete balance, but on similarity of fate of dissolved Mg across treatments, irrespective of its origin (olivine or kieserite). We applied Method 2 to three response variables (*X* in Eq.3): Mg concentration in plant biomass, total Mg offtake in plant biomass, and bioavailable soil Mg.

### Statistical analysis

Analysis of variance was used to analyse the randomized block experiment. Residual plots revealed that for most response variables a log-transformation stabilized the variances. Therefore all response variables, except soil water pH, were logarithmically transformed prior to analysis. Each pair of treatments means was tested for significance at the 1% level using a Student t-test which employs the residual mean square of the analysis of variance as an estimate of the variance, see e.g. [12]. The results of these pairwise tests are presented by annotating mean values, given on the original scale, with a superscript letter such that treatments sharing the same letter are not significantly different at the 1% level, while treatment means with no letter in common are significantly different. We verified that only rare and minor discrepancies occurred between statistical analysis on the original and on the log-scale.

Harvested biomass and nutrient concentrations in plant tissue were measured per separate harvest event. Their values were first aggregated to total biomass yield and to mass-weighted average nutrient concentrations, prior to statistical analysis.

### Olsen model for olivine weathering

Estimates of weathering from our pot experiment were compared with reaction kinetics based on laboratory measurements under a wider range of conditions. We used a simple model based on [10], [13], expressing olivine weathering per unit area of crystal surface in terms of the ‘carbonation rate’ *r* (mol m^−2^ s^−1^):
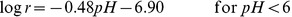
(4a)

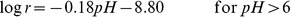
(4b)


These regression equations were based by [10] on geometrical surface area for spherical particles, as opposed to BET surface area. (The two approaches give different estimates of the regression parameters, see [10] for a comparative study on a large set of laboratory data.) The dependence on temperature (*T*) is given by the Arrhenius equation:
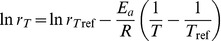
(5)with *T*_ref_ for reference temperature (298 K), and *E*_a_ for activation energy (63 kJ/mol; [14]). *R* is the Universal gas constant (8,31 J K^−1^ mol^−1^) and *r_T_*_ref_ is the carbonation rate at reference temperature. Using 4.4 10^−5^ m^3^ mol^−1^ for the molar volume of olivine, *V*_m_ [15], the weathering rate per unit surface area is converted into a corresponding retreat of the reactive surface position (‘shrinking particle model’). Following [14], the model thus accounts for the time *t*_d_ that is required to completely dissolve a particle, in function of its size class. For particles of diameter *D*_0_ (m), *t*_d_ (s) is approximated [10] as
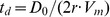
(6)To implement the calculations, we expressed the particle size distribution in 95 size classes. The model was applied at the lowest and highest soil water pH observed in our Control treatment, respectively. Model results are given as total mass fraction weathered, as well as contributions from selected particle size classes.

## Results

### Plant biomass and plant analysis

Plant growth did not differ from the Control in any treatment, except OLIV4 which gave a 15.6% higher dry matter (DM) yield (Table S5; Treatment codes in Table 1). Expressed per unit pot surface area, DM yields corresponded to 19.2 to 23.6 10^3^ kg ha^−1^. These values are roughly 50% higher than typically obtained under field conditions, light interception in pot culture being larger than in the field. Total growth duration, however, was comparable to field conditions in North-West Europe. The yields obtained are proof of vigorous growth in all treatments.

Element concentrations in plant dry matter are given in Table 2. N concentration was not affected by olivine addition. Values are considered low - relative to values between 25 and 35 g kg^−1^ for normal production conditions. They show that fertiliser N application at the equivalent of 480 kg N ha^−1^ (including topdressing after each harvest) was modest, relative to crop demand. P concentrations were within the range of 3.0 to 4.5 g kg^−1^ typically found, and were somewhat reduced by olivine. Total P uptake, however, was not. K uptake, in contrast, was increased by olivine, but only at its highest dose (OLIV4). Extra K uptake by plants was possibly due to preferential adsorption of Mg^++^ (over K^+^) on the soil complex, releasing K^+^ into solution. Mg, Si and Ni concentrations in plant dry matter were higher than in the Control, even at the smallest olivine dose, and effects increased with higher doses. Ca, in contrast, decreased with larger olivine doses. This is attributed to competition between Ca and Mg uptake, and shows that olivine might induce a nutritional imbalance as it does in natural systems with high inherent Mg/Ca ratios [16], [17], [18], [19]. There was a significant rise in Ni concentration in grass at all doses of olivine, with a fivefold increment from 531 (Control) to 2669 µg per kg dry biomass (OLIV4).

**Table 2:** Element concentrations^1^ in plant dry matter, mass-weighted average over all six harvests.

Treatment	N	P	K	Mg	Ca	Si	Ni
	g kg^−1^	g kg^−1^	g kg^−1^	g kg^−1^	g kg^−1^	g kg^−1^	µg kg^−1^
Control	19.32^a^	3.556^c^	24.77^a^	2.127^a^	4.65^d^	2.47^a^	531^a^
KIES1	19.18^a^	3.474^bc^	25.09^a^	2.414^bc^	4.18^c^	2.69^a^	475^a^
KIES2	19.27^a^	3.591^c^	25.09^a^	2.594^c^	3.75^b^	2.73^a^	517^a^
OLIV1	18.75^a^	3.469^bc^	24.32^a^	2.406^b^	4.34^cd^	3.72^b^	696^b^
OLIV2	18.91^a^	3.332^ab^	24.36^a^	2.517^bc^	4.19^c^	4.37^bc^	955^c^
OLIV3	18.10^a^	3.237^a^	24.95^a^	2.549^bc^	3.68^b^	5.05^c^	1552^d^
OLIV4	18.01^a^	3.183^a^	28.92^b^	3.072^d^	1.94^a^	6.98^d^	2669^e^

1 *Treatment means sharing the same letter within a column are not significantly different at the 1% level according to a pairwise t-test, while treatment means with no letter in common are significantly different.*

### Soil analysis

Bioavailability of elements in soil at the end of the experiment is listed in Table 3, only for elements that showed significant responses. Available soil Mg, Si, and Ni, and soil pH were all increased by olivine, and effects increased with larger doses. Soil pH increased from 4.89 in the Control to 5.96 at the largest olivine dose. While changes in Mg, Si and Ni may directly reflect the addition of these elements (with olivine), some other elements were affected, too. Available K increased while total soil K remained unaffected. Available P decreased slightly with higher olivine dose, but only in one extractant (0.01 M CaCl_2_, Table 3), not in ammoniumlactate-acetic acid (P_AL_). (P_AL_ in the Control was 24.8 mg per 100 g soil). These responses were possibly related to changes in soil pH. Inorganic soil N was not affected. Its value in the Control treatment was 15.00 mg per kg dry soil.

**Table 3:** Bioavailability^1^ of elements in soil at the end of the experiment.

Treatment	pH	P	K	Mg	S	Si	Ni
		mg kg^−1^	mg kg^−1^	mg kg^−1^	mg kg^−1^	mg kg^−1^	µg kg^−1^
Control	4.89^a^	1.95^b^	16.30^a^	36.30^a^	6.69^ab^	14.00^a^	90^a^
KIES1	4.90^a^	1.75^ab^	20.80^ab^	42.00^b^	6.99^ab^	18.59^ab^	78^a^
KIES2	5.04^ab^	1.55^a^	25.28^b^	47.40^b^	8.13^b^	16.37^ab^	70^a^
OLIV1	4.99^ab^	1.95^b^	19.73^ab^	61.30^c^	5.49^a^	16.65^ab^	130^b^
OLIV2	5.10^b^	2.00^b^	18.85^ab^	84.50^d^	5.81^a^	15.90^ab^	260^c^
OLIV3	5.34^c^	1.60^a^	25.12^b^	129.80^e^	6.33^ab^	19.32^b^	598^d^
OLIV4	5.96^d^	1.575^a^	37.55^c^	287.2^f^	7.73^b^	32.08^c^	1383^e^

1 *Treatment means sharing the same letter within a column are not significantly different at the 1% level according to a pairwise t-test, while treatment means with no letter in common are significantly different.*

Increments in total soil Mg and total soil Ni reflect the total amounts of olivine added, because all olivine was dissolved by sample destruction with *Aqua regia*. For these and other elements with significant responses, total concentrations in soil are given in Table S6. Total N, Ca, K and S in soil were not affected by olivine. Their values in the Control treatment were 1.15 g N, 923 mg Ca, 355 mg K, and 198 mg S per kg dry soil.

### Soil water analysis

Element concentrations in soil water were measured at two occasions in selected treatments (Table S7). Concentrations of Mg, Ni and Si in water were higher in OLIV2, OLIV3 and OLIV4 than in the Control. Soil water pH and alkalinity were measured in selected treatments (Table 4). All values refer to soil water at a harvest event (grass cutting), prior to dressing of new fertiliser for regrowth. Throughout the experiment, pH and alkalinity were higher in OLIV4 than in the Control treatment. Only on April 7 were soil water pH and alkalinity measured in all treatments. Soil water pH, then, was increased only in OLIV4, while alkalinity was increased in OLIV3 and OLIV4. The fluctuations in pH and alkalinity over time, and the absence of pH and alkalinity responses at lower olivine doses, are not fully understood. They may reflect buffering by the soil system. Also, the application of fertilisers, root activity, and fluctuations in soil water content affect the inorganic C balance. For these reasons we did not attempt to estimate olivine weathering or net CO_2_ sequestration from changes in alkalinity.

**Table 4:** Soil water pH^1^ and alkalinity^1^.

	Oct.29	Nov.26	Jan.5	Feb.17	Apr.7
**Treatment**	**Soil water pH**				
Control	5.40^a^	5.68^a^	6.52^a^	6.65^a^	6.35^a^
KIES1					6.70^a^
KIES2				6.32^a^	6.68^a^
OLIV1					6.54^a^
OLIV2				6.37^a^	6.27^a^
OLIV3					6.79^a^
OLIV4	7.04^b^	7.21^b^	6.81^b^	6.98^a^	7.55^b^
	**Soil water alkalinity (mmol/l)**				
Control	0.23^a^	0.53^a^	1.26^a^	1.40^a^	0.55^a^
KIES1					0.85^ab^
KIES2				2.37^ab^	1.63^bc^
OLIV1					0.79^ab^
OLIV2				2.01^ab^	0.68^ab^
OLIV3					1.95^bc^
OLIV4	7.23^b^	9.58^b^	5.92^b^	3.46^b^	2.91^c^

1 *Treatment means sharing the same letter within a column are not significantly different at the 1% level according to a pairwise t-test, while treatment means with no letter in common are significantly different.*

### Olivine weathering in the experiment

Olivine weathering estimated by Method 1 is shown in Table 5. At the lowest olivine dose (OLIV1), 14.8% of applied olivine Mg became bioavailable during the 32 weeks of the trial. This fraction decreased steeply with higher olivine doses. According to Method 2 (Table 6), estimates of olivine weathered in treatment OLIV1 ranged from 13% to 58%, depending on the indicator chosen. The lower value refers to Mg content in plant biomass, the upper value to bioavailable soil Mg.

**Table 5:** Magnesium (Mg) balance terms, and ratio of bioavailable to applied Mg (Method 1).

	(a)	(b)	(c)	(d)	(e)
Treatment	Mg applied	Mg in biomass	Bioavailable Mg in soil	Increment over control	Ratio^1^ of bioavailable to applied Mg
	g/pot	g/pot	g/pot	g/pot	
Control	0.000	0.213	0.363	0.000	
KIES1	0.243	0.231	0.420	0.075	30.9%
KIES2	0.486	0.245	0.474	0.143	29.4%
OLIV1	1.88	0.241	0.613	0.278	14.8%
OLIV2	9.40	0.258	0.845	0.527	5.6%
OLIV3	47	0.261	1.298	0.983	2.1%
OLIV4	235	0.355	2.872	2.651	1.1%

1 *For the olivine treatments (OLIV1-4), the fraction weathered (F_weath_) is estimated by Method 1 as this ratio. See text,* *Eq.2* *. For kieserite, this ratio suggest that only part of dissolved Mg was retrieved, see* *Discussion* *.*

**Table 6:** Fraction of olivine weathered in OLIV1, estimated via Method 2 (similarity with kieserite).

	(a)	(b)	(c)	(d)	(e)	(f)
Treatment	Control	KIES1	KIES2	OLIV1	Kieserite equivalent^1^ (g Mg/pot)	Fraction^2^ olivine weathered
Mg applied (g/pot)	0	0.243	0.486	1.88		
*Indicator variable:*						
[Mg] in biomass (g/kg)	2.127	2.414	2.594	2.406	0.236	13%
Mg offtake (g/pot)	0.213	0.231	0.245	0.241	0.416	22%
Bioavail.soil Mg (mg/kg)	36.3	42.0	47.4	61.3	1.10	58%

1 *Kieserite equivalent (e) is the dose of magnesium (Mg, g/pot) in kieserite form, required to achieve the same effect on Mg-indicators (first column) as the effect observed in OLIV1 (column d).*

2 *Fraction weathered in OLIV1 calculated as (e)/1.88, where 1.88 is the Mg dose given as olivine in OLIV1. See text,* *Eq. 3* *.*

## Discussion

### Estimation of olivine weathering

Method 1 presumes that all Mg released from olivine is retrieved in soil and plant sampling. This, however, is challenged by the only partial retrieval of Mg from kieserite (30%, Table 5). While kieserite is considered highly soluble, Mg from kieserite then must have precipitated in a form not extracted as bioavailable (in 0.01 M CaCl_2_); or it was lost from the system. The former option seems more likely, as cations are generally held by the soil's negatively charged adsorption complex and, moreover, we aimed to avoid net water percolation. The same fate, then, may have applied to Mg released from olivine, and so our estimation of weathering by Method 1 may be too low. (Amounts of Mg in soil water are ignored as a balance term. These were two orders of magnitude smaller than bioavailable soil Mg; divalent cations are strongly adsorbed by soil). For Method 2, while the two lower values in Table 6 (column f) were obtained by interpolation within the data range, the highest value was extrapolated beyond the Mg dose of KIES2. Without extrapolation, the equivalency of the olivine dose in OLIV1 must be set to 0.486 g kieserite-Mg pot^−1^ (as in KIES2), and the corresponding estimate of the fraction weathered becomes 26%.

### Weathering in experiment versus Olsen model

The Olsen model indicated that for our experiment, with monthly average temperatures as measured and with pH fixed at 5.4, a skin of thickness 0.12 µm on olivine particles would react during the trial. Model outcomes are independent of olivine dose. Given the particle size distribution of our olivine product (Fig. S1), 0.12 µm corresponds to a mass fraction of 9.0% of olivine applied. At pH = 6.7, the model predicted a layer of thickness 0.04 µm, or 4.0 mass%. Disproportionality between skin thickness and mass weathered is due to complete consumption of very fine particles. At pH 5.4, the model predicted that particles smaller than 2 µm contributed 56% of total weathering, particles smaller than 20 µm contributed 90%. At pH 6.7, these fractions were 66% and 93%, respectively, according to the model. Above model estimates for overall weathering (all particle sizes) at both pH values are below our experimental value for OLIV1 (Tables 5, 6), and are around our value for OLIV2 (Table 5). We conclude that modelled and measured weathering rates differ by less than one order of magnitude for the lower doses. But also that negative feedback occurred at high olivine doses in soil. As stated, the model ignores this and thus overestimates weathering at high olivine doses, under our conditions. Olsen's model was based on a compilation of laboratory data for (sometimes pre-treated) olivine grains in stirred buffer solutions at various pH; the contrasting conditions complicate a direct comparison with our data. Feedbacks in soil are possibly due to changes in boundary layer pH, high aqueous Si concentration, or the formation of a passivating silica layer on olivine particles [10], [20].

The model indicates that, at our particle size distribution, particles smaller than 20 µm accounted for about 90% of all weathering during the trial. If this is true, then a drop in weathering rate must be expected over longer periods as fine particles are consumed. On the other hand, we do not know the impact on olivine particles of prolonged exposure to the soil environment. Also, the large discrepancy between measured BET surface of our olivine product (4.8 m^2^ g^−1^), and surface area calculated from measured particle size distribution (0.39 m^2^ g^−1^ for spherical particles) warrants caution in extrapolating the model predictions over longer periods. The high ratio between the two estimators of specific surface area may indicate micro-porosity or micro-cracks, phenomena recently documented for olivine [21].

### Carbon sequestration potential

Gross sequestration amounts to 1.2 kg CO_2_ per kg of olivine weathered (Eq. 1), and we use this figure to estimate gross CO_2_ sequestration associated with weathering in our experiment. By our most conservative estimate of weathering (14.8%), gross sequestration at the dose of 8 g olivine per pot (1630 kg ha^−1^, a dust layer of about 0.1 mm thickness) was equivalent to 29 10^3^ kg CO_2_ km^−2^ during the course of the experiment. (This is calculated as 0.148 * 1630 kg ha^−1^ * 1.2 kg CO_2_ per kg olivine = 290 kg ha^−1^ = 29 10^3^ kg km^−2^.) The amount of olivine weathered, and hence of gross CO_2_ sequestration, roughly doubled for each fivefold increment of olivine applied. (Multiply fraction weathered from Table 5 with corresponding olivine dose, Table 1.) At the highest dose (OLIV4), gross sequestration as calculated by the same rule (1.2 kg kg^−1^) was 269 10^3^ kg CO_2_ km^−2^. To put these figures into perspective: if 29 to 269 10^3^ kg CO_2_ km^−2^ would be sequestered annually on the world's entire agricultural area, this would correspond to 1.5 to 13.9 Pg CO_2_ a^−1^ or 4.7% to 43.6% of the annual global CO_2_ release from combustion of fossil fuels (emission data 2008 in [22]). Our values can also be compared to annual CO_2_ sequestration by natural silicate weathering as calculated for catchments. The global average is estimated at 1.9 10^3^ kg C (7.0 10^3^ kg CO_2_) km^−2^a^−1^, based on the GEM-CO_2_ model [23]. Values below 5 10^3^ kg CO_2_ km^−2^a^−1^ were listed for acidic formations in the humid tropics [24], whereas the basaltic Deccan Traps would capture some 55 10^3^ kg CO_2_ km^−2^a^−1^ [25]. As an average for the volcanic Japanese Archipelago [26], reports an intermediate value of 6.05 10^3^ kg C (about 22 10^3^ kg CO_2_) km^−2^a^−1^. So, gross CO_2_ sequestration at our lowest dose was four times larger than the annual global average, and about 30% higher than the annual Japanese average. Gross sequestration in our OLIV2 treatment (54.7 10^3^ kg km^−2^ at olivine dose of 8150 kg ha^−1^) would be similar to the high extreme given for the Deccan Traps. This is still far below extreme rates reported for carbonation of certain mine wastes [27].

Extrapolated to the world's agricultural area, our lower and upper olivine doses would correspond to global olivine inputs of 8 and 1000 Pg. For comparison: annual global hard coal production in 2011 was about 6.2 Pg [28]. Further extrapolation must take into account that feedbacks in oceanic carbonate chemistry would reduce CO_2_ sequestration efficiency by some 20% according to [6]; but also that, once applied, olivine would continue to sequester CO_2_ during many years, if our relative weathering rates (Table 5) remained valid over longer periods. Net sequestration will be smaller than gross sequestration, as part of the carbon captured as bicarbonate by ‘enhanced weathering’ may first be neutralized by soil acids, to escape as CO_2_ back into the atmosphere. Of course, this is no zero-effect operation because the neutralisation of soil acidity would otherwise have required fossil carbonates, releasing CO_2_ into the atmosphere. Indeed, basic silicates can be used as an alternative for the common practice of liming to counter soil acidification. Contrary to liming with chalk, there is no net release of CO_2_ here. Further, estimating net sequestration potential requires accounting for CO_2_ equivalents spent in mining, transporting and milling olivine rock. According to [29], the total energy required for crushing and milling, including tertiary (ultra-fine, 10 µm) milling, corresponds to an emission of 174 g CO_2_ per kg CO_2_ sequestered, far more important than the CO_2_ cost for mining and transportation. This value was calculated by [29] from primary sources [30], [31].

### Notes on olivine application to agricultural systems

It is difficult to anticipate all impacts that input of fresh basic silicates may have on agricultural systems. Impacts are likely to vary widely depending on soil, crop and climate characteristics. Based on Eq. 1 as well as our observations (Tables 3, 4) it can be expected that massive application of fast-weathering silicates will induce changes in soil pH and associated chemistry, and this may affect the availability of plant nutrients. Mg-induced Ca deficiency, for example, is well known from non-adapted crop species on serpentine-derived soils [32]. Such deficiencies could possibly be corrected in well-managed high input systems, but may pose problems in some extensively managed systems. Other aspects that warrant closer inspection are (a) the possible enhancement of soil organic matter decomposition which might result in loss of soil quality and net CO_2_ emission (liming of acidified soil is known to reduce soil organic carbon stocks); and (b) the possible fixation of phosphates on freshly formed Fe-complexes, if silicates rich in Fe are used.

Finally, the relatively fast release of bioavailable Ni from olivine into the food chain and the wider environment could set limits to permissible olivine doses. While our experiment revealed no negative impacts on plant growth, it seems not currently possible to set general no-effect thresholds. Bioavailability of Ni, and toxicity to plants of Ni in soils amended with soluble metal salts were studied by [33], [34], [35]. Those studies underline that while soil pH is key to bioavailability, thresholds for toxicity depend strongly on other characteristics such as cation exchange capacity (CEC) and, within the acidic pH range, organic matter content. The study by [33] showed that the growth of oats (*Avena sativa* L.) seedlings was reduced by 50% at thresholds for total Ni in soil that varied, depending on soil properties, from 41 to 1321 mg kg^−1^, with lowest values for sandy soils. Ranges by [34], who used barley (*Hordeum vulgare L.*) root growth and tomato (*Solanum lycopersicum L.*) shoot growth to express Ni-induced inhibition, are of the same order of magnitude. For comparison, our maximum total soil Ni content was 284 mg kg^−1^ in OLIV4 (Table S6), but this included all Ni in unweathered olivine, too. (Weathering in OLIV4 was estimated at 1.1% only; Table 5.) Bioavailable Ni in this treatment was 1.38 mg kg^−1^, and had no negative impact on growth (to the contrary). Although all Ni in olivine added to soils will ultimately be released from the silicate, this does not necessarily imply a build-up of bioavailable or phytotoxic Ni. Toxicity thresholds in ‘aged soils’ (18 months after amendment with soluble Ni salts) were shown to be up to 100 times larger than in freshly amended soils [35], suggesting immobilisation of previously bioavailable Ni as time proceeds.

Ni effects on plant growth were also studied in areas that are naturally rich in Ni derived from igneous bedrock. Trace metals in pastures over basalts were studied by [36] in the French Massif Central. They reported total Ni in soil between 168 and 214 mg kg^−1^, and values of around 2.17 mg kg^−1^ for Ni in aboveground plant biomass. The latter is close to 2.67 mg kg^−1^ found at the highest dose (OLIV4) in our trial. These are well below the threshold of 10 mg kg^−1^ for Ni toxicity in plants according to [37]. Higher levels (11.1–39.3 mg kg^−1^ in forage) were reported by [32] for pastures on serpentine-derived soils in Galicia, Spain. These were associated with high Ni accumulation in kidney tissue of grazing cattle, while Ni in liver and muscle tissues remained undetectable. For the same area in northern Spain, [38] reported Ni contents of 12 to 34 mg kg^−1^ in foliage of various crops, with highest values for sugar beet (*Beta vulgaris* L.), but they found no indications of Ni toxicity. Growth inhibition was demonstrated for ryegrass (*Lolium perenne* L.), at shoot Ni concentrations of about 100 mg kg^−1^ [39]. In summary, while toxicity of Ni to plants and animals remains undisputed, evidence in the cited studies occurred at much higher concentrations than those found in our trial at high olivine doses. Multi-annual studies on olivine application to different types of crops and soils are needed for a more comprehensive assessment of the risks associated with Ni from olivine. The use of other fast weathering basic silicates, low in heavy metals, might waive this issue, but such minerals are less abundant than olivine.

### Conclusions

The weathering rate of finely ground olivine in our soil was substantial. Between 13% and 58% of added olivine weathered during the 32 weeks of the trial, depending on the method of estimation. This range applies to our lowest olivine dose only, and is higher than estimates made by the Olsen model, which is based on laboratory conditions. At higher doses, however, weathering (relative to dose applied) decreased steeply in our experiment, and was less than predicted by the model. This suggests negative feedbacks for weathering in the soil environment, unaccounted for by the model.

Olivine increased soil water pH and alkalinity, plant uptake of Mg, Si and Ni, as well as bioavailability of Mg and Ni in soil. Olivine clearly suppressed Ca uptake which is attributed to competition by Mg. All these effects increased with higher olivine doses. There was a slight negative effect on P content in plant biomass at high olivine doses. At the highest dose (204 10^3^ kg ha^−1^), olivine increased plant biomass (+15.6%) as well as K concentration in plant biomass (+16.5%). The cause of increased growth remains unclear, and may at our plant nutrient levels be unrelated to increased K uptake.

The appreciable weathering rate and lack of evidence for negative impacts on plant growth support the feasibility of the ‘enhanced weathering’ concept. Yet, massive application of olivine in agriculture may cause imbalances in plant nutrition, notably at low Ca availability, and will bring Ni into the food chain. In our case, olivine increased grass Ni concentration already at the dose of 1630 kg olivine ha^−1^, from 0.531 to 0.696 mg kg^−1^. At the extreme olivine dose of 204*10^3^ kg ha^−1^, grass Ni concentration was 2.67 mg kg^−1^. Although this is below a phytotoxic threshold of 10 mg kg^−1^, it implies that the use of olivine in agricultural systems must remain within certain limits. Long term field studies are required to assess such limits under different climatic, soil and crop conditions.

## Supporting Information

Figure S1 **Particle size distribution of the olivine product used.**
(DOCX)

Figure S2 **Temperature regime (outdoors period of the experiment).**
(DOCX)

Figure S3 **Temperature regime (greenhouse period of the experiment).**
(DOCX)

Table S1 **Chemical composition of the olivine product used.**
(DOCX)

Table S2 **Chemical soil characteristics before experiment.**
(DOCX)

Table S3 **Fertiliser applications.**
(DOCX)

Table S4 **Soil extraction methods; and plant and soil analysis methods.**
(DOCX)

Table S5 **Harvested plant biomass.**
(DOCX)

Table S6 **Total element concentrations in soil (Aqua regia), at last harvest.**
(DOCX)

Table S7 **Concentrations of Ca, Mg, Ni, and Si in soil water.**
(DOCX)

## References

[1] United Nations Framework Convention on Climate Change (UNFCCC) (1992) United Nations. FCCC/informal/84 ge.05-62220 (e) 200705.

[2] Lal R (2008) Carbon sequestration. Philos Trans R Soc Lond B Biol Sci 363: 815–830. 17761468 10.1098/rstb.2007.2185 PMC2610111

[3] Smith P, Martino D, Cai Z, Gwary D, Janzen H, et al (2008) Greenhouse gas mitigation in agriculture. Phil Trans R Soc B 363: 789–813. 17827109 10.1098/rstb.2007.2184 PMC2610110

[4] Dunsmore HE (1992) A geological perspective on global warming and the possibility of carbon dioxide removal as calcium carbonate mineral. Energy Conversion and Management 33: 565–572.

[5] Schuiling RD, Krijgsman P (2006) Enhanced Weathering: An Effective and Cheap Tool to Sequester CO_2_ . Climatic Change 74: 349–354.

[6] Koehler P, Hartmann J, Wolf-Gladrow DA (2010) Geoengineering potential of artificially enhanced silicate weathering of olivine. Proc Natl Acad Sci USA 107: 20228–20233. 21059941 10.1073/pnas.1000545107 PMC2996662

[7] Hartmann J, Kempe S (2008) What is the maximum potential for CO_2_ sequestration by “stimulated” weathering on the global scale? Naturwissenschaften 95: 1159–1164. 18754090 10.1007/s00114-008-0434-4

[8] FAOSTAT, Available: http://faostat.fao.org/site/377/default.aspx#ancor accessed 19 May 2011.

[9] MacIntire W, Marshall H (1959) Fertilizer Raw Materials - Magnesium Ammonium Phosphate From Olivine and Rock Phosphate. Journal of Agricultural and Food Chemistry 7: 566–568.

[10] Olsen AA (2007) Forsterite Dissolution Kinetics Applications and implications for chemical weathering, Dissertation Virginia Polytechnic Institute. Available: http://scholar.lib.vt.edu/theses/available/etd-07052007-135551/

[11] Gregg SJ, Sing KSW (1982) *Adsorption, Surface Area and Porosity*, 2nd ed., Academic Press, London.

[12] Snedecor GW, Cochran WG (1967). Statistical methods, sixth edition. Iowa State University Press. Ames, Iowa, USA.

[13] TNO (2008) TNO-U-R-0776/B Desk study on the feasibility of CO_2_ sequestration by mineral carbonation. http://www.rijksoverheid.nl/bestanden/documenten-en-publicaties/kamerstukken/2009/01/26/reactie-op-brief-van-hr-schuiling-over-olivijn-rapport/kl2009006101aolivijnrapport.pdf

[14] Olsen AA, Rimstidt JD (2007) Using a mineral lifetime diagram to evaluate the persistence of olivine on Mars. American Mineralogist 92: 598–602.

[15] Lasaga AC (1998) Kinetic Theory in the Earth Sciences 728 p. Princeton University Press Princeton, New Jersey.

[16] Asemaneh T, Ghaderian SM, Baker AJM (2007) Responses to Mg/Ca balance in an Iranian serpentine endemic plant, Cleome heratensis (Capparaceae) and a related non-serpentine species, C. foliolosa. Plant and Soil 293: 49–59.

[17] Lazarus BE, Richards JH, Claassen VP, O'Dell RE, Ferrell MA (2011) Species specific plant-soil interactions influence plant distribution on serpentine soils. Plant and Soil 342: 327–344.

[18] Nagy L, Proctor J (1997) Plant growth and reproduction on a toxic alpine ultramafic soil: Adaptation to nutrient limitation. New Phytologist 137: 267–274. 10.1046/j.1469-8137.1997.00799.x 33863177

[19] Samecka-Cymerman A, Garbiec K, Kolon K, Kempers AJ (2009) Factor analysis of the elemental composition of Pteridium aquilinum from serpentine and granite soils as a tool in the classification of relations between this composition and the type of parent rock in the Śle{ogonek}ża Massif in Lower Silesia, Poland. Environmental Geology 58: 509–514.

[20] Béarat H, McKelvy MJ, Chizmeshya AVG, Gormley D, Nunez R, et al (2006) Carbon Sequestration via Aqueous Olivine Mineral Carbonation: Role of Passivating Layer Formation. Environ Sci Technol 40: 4802–4808. 16913142 10.1021/es0523340

[21] Kwon S, Fan MH, DaCosta HFM, Russell AG (2011) Factors affecting the direct mineralization of CO2 with olivine. Journal of Environmental Sciences-China 23: 1233–1239. 22128528 10.1016/s1001-0742(10)60555-4

[22] Quéré C le, Raupach MR, Canadell JG, Marland G, Bopp L, et al (2009) Trends in the sources and sinks of carbon dioxide. Nature Geoscience 2: 831–836.

[23] Suchet PA, Probst JL, Ludwig W (2003) Worldwide distribution of continental rock lithology: Implications for the atmospheric/soil CO_2_ uptake by continental weathering and alkalinity river transport to the oceans. Global Biogeochemical Cycles 17: 7–1.

[24] Mora A, Alfonso JA, Baquero JC, Balza L, Pisapia D (2010) Caura River basin: Weathering rates, CO_2_ consumption, and chemistry of major and trace elements in an Orinoco River tributary coming from the Precambrian Guayana Shield, Venezuela. Geochemistry, Geophysics, Geosystems 11.

[25] Dessert C, Dupré B, François LM, Schott J, Gaillardet J, et al (2001) Erosion of Deccan Traps determined by river geochemistry: Impact on the global climate and the 87Sr/86Sr ratio of seawater. Earth and Planetary Science Letters 188: 459–474.

[26] Hartmann J (2009) Bicarbonate-fluxes and CO_2_-consumption by chemical weathering on the Japanese Archipelago - Application of a multi-lithological model framework. Chemical Geology 265: 237–271.

[27] Wilson SA, Dipple GM, Power IM, Thom JM, Anderson RG, et al (2009) Carbon Dioxide Fixation within Mine Wastes of Ultramafic-Hosted Ore Deposits: Examples from the Clinton Creek and Cassiar Chrysotile Deposits, Canada. Economic Geology 104: 95–112.

[28] World Coal Association, Available: http://www.worldcoal.org/coal/coal-mining/, accessed on May 22, 2012.

[29] Hangx SJT, Spiers CJ (2009) Coastal spreading of olivine to control atmospheric CO_2_ concentrations: a critical analysis of viability. International Journal of Greenhouse Gas Control 3: 757–767.

[30] CIPEC (2005) Benchmarking the Energy Consumption of Canadian Open-pit Mines. Mining Association of Canada and Natural Resources Canada, pp. 56.

[31] O'Connor WK, Dahlin DC, Rush GE, Gerdemann SJ, Penner LR, et al.. (2005) Aqueous Mineral Carbonation: Mineral Availability, Pretreatment, Reaction Parametrics, and Process Studies. , DOE/ARC-TR-04-002, p. 20.

[32] Miranda M, Benedito JL, Blanco-Penedo I, López-Lamas C, Merino A, et al (2009) Metal accumulation in cattle raised in a serpentine-soil area: relationship between metal concentrations in soil, forage and animal tissues. J. of Trace Elements in Medicine and Biology 23: 231–238. 10.1016/j.jtemb.2009.03.004 19486833

[33] Weng L, Wolthoorn A, Lexmond TM, Temminghoff EM, Riemsdijk WH van (2004) Understanding the Effects of Soil Characteristics on Phytotoxicity and Bioavailability of Nickel Using Speciation Models. Environ Sci Technol 38: 156–162. 14740731 10.1021/es030053r

[34] Rooney CP, Zhao F-J, McGrath SP (2007) Phytotoxicity of nickel in a range of European soils: Influence of soil properties, Ni solubility and speciation. Environmental Pollution 145: 596–605. 16733077 10.1016/j.envpol.2006.04.008

[35] Smolders E, Oorts K, Sprang P van, Schoeters I, Janssen CR, et al (2009) Toxicity of trace metals in soil as affected by soil type and aging after contamination: Using calibrated bioavailability models to set ecological soil standards. Environmental Toxicology and Chemistry 28: 1633–1642. 19301943 10.1897/08-592.1

[36] Neel C, Soubrand-Colin M, Piquet-Pissaloux A, Bril A (2007) Mobility and bioavailability of Cr, Cu, Ni, Pb and Zn in a basaltic grassland: comparison of selective extractions with quantitative approaches at different scales. Applied Geochemistry 22: 724–735.

[37] Kabata-Pendias A (2001) Trace elements in soil and plants. 3^rd^ ed. USA, CRC Press.

[38] Fernández S, Seoane S, Merino A (1999) Plant heavy metal concentrations and soil biological properties in agricultural serpentine soils. Communications in Soil Science and Plant Analysis 30: 1867–1884.

[39] Dijkshoorn W, Broekhoven LW van, Lampe JEM (1979) Phytotoxicity of zinc, nickel, cadmium, lead, copper and chromium in three pasture plant species supplied with graduated amounts from the soil. Neth J Agric Sci 27: 241–253.

