# Supplementary material for: Olivine Weathering in Soil, and Its Effects on Growth and Nutrient Uptake in Ryegrass (Lolium perenne L.): A Pot Experiment
Source: PLoS One. 2012 Aug 9;7(8):e42098. doi: 10.1371/journal.pone.0042098 (PMC3415406; doi:10.1371/journal.pone.0042098)
Supplement: Figure S1 — Particle size distribution of the olivine product used. (DOCX) [file pone.0042098.s001.docx]

*Figure S1. Particle size distribution of North Cape olivine sand.*

*Optical analysis by LS230 Fluid Module, Fraunhofer.*
