# Supplementary material for: Olivine Weathering in Soil, and Its Effects on Growth and Nutrient Uptake in Ryegrass (Lolium perenne L.): A Pot Experiment
Source: PLoS One. 2012 Aug 9;7(8):e42098. doi: 10.1371/journal.pone.0042098 (PMC3415406; doi:10.1371/journal.pone.0042098)
Supplement: Figure S3 — Temperature regime (greenhouse period of the experiment). (DOCX) [file pone.0042098.s003.docx]

*Figure S3. Daily minimum, maximum and mean temperature during the greenhouse period of the experiment. Wageningen, The Netherlands, 2009-2010.*
