# Supplementary material for: Olivine Weathering in Soil, and Its Effects on Growth and Nutrient Uptake in Ryegrass (Lolium perenne L.): A Pot Experiment
Source: PLoS One. 2012 Aug 9;7(8):e42098. doi: 10.1371/journal.pone.0042098 (PMC3415406; doi:10.1371/journal.pone.0042098)
Supplement: Table S1 — Chemical composition of the olivine product used. (DOCX) [file pone.0042098.s004.docx]

*Table S1. Chemical composition^1^ of ‘North Cape olivine sand’ by Sibelco Nordic.*

|  | Content  (mg per kg olivine) |
| --- | --- |
| Ca | 488 |
| Fe | 40153 |
| K | 1659 |
| Mg | 234881 |
| Mg (bioavailable) | 204 |
| Ni | 2445 |
| Ni (bioavailable) | 0.020 |

*^1.^All based on destruction with Aqua Regia (HNO3-HCl), except bioavailable Mg and Ni which were extracted in 0.01 M CaCl2.*
