# Supplementary material for: Olivine Weathering in Soil, and Its Effects on Growth and Nutrient Uptake in Ryegrass (Lolium perenne L.): A Pot Experiment
Source: PLoS One. 2012 Aug 9;7(8):e42098. doi: 10.1371/journal.pone.0042098 (PMC3415406; doi:10.1371/journal.pone.0042098)
Supplement: Table S2 — Chemical soil characteristics before experiment. (DOCX) [file pone.0042098.s005.docx]

*Table* *S2. Chemical soil characteristics before experiment.*

| **Element** | **destruction**  **HNO_3_-HCl  (Aqua Regia)** | **extraction**  **0.43 M HNO_3_** | **extraction**  **0.01 M CaCl_2_** |
| --- | --- | --- | --- |
|  |  |  |  |
|  | ***ICP-AES Thermo*** | ***ICP-AES Thermo*** | ***ICP-AES Varian*** |
|  | mg kg^-1^ | mg kg^-1^ | mg kg^-1^ |
| Al | 3827 | 786 |  |
| As | 3.74 |  |  |
| Ca | 884 | 632 |  |
| Fe | 5096 | 1493 |  |
| K | 395 | 75.9 | 41.7 |
| Mg | 381 | 51.6 | 44.4 |
| Mn | 225 | 99.0 | 9.22 |
| Na | 33 |  |  |
| P | 716 | 475 | 3.01 |
| Zn | 26.6 | 11.3 | 3.184 |
|  |  |  |  |
|  | ***ICP-MS*** | ***ICP-MS*** | ***ICP-MS*** |
|  | mg/kg | mg/kg | µg/kg |
| As |  | 1.78 | 13.5* |
| Ba | 21.3 | 13.8 | 1037* |
| Cd | 0.24 | 0.24 | 77.3* |
| Co | 1.17 | 0.48 | 20.8* |
| Cr | 11.8 | 2.04 | 6.06* |
| Cu | 9.05 | 7.13 | 65.0* |
| Ni | 3.74 | 0.68 | 98.3* |
| Pb | 16.1 | 13.4 | 6.95* |
|  |  |  |  |
| pH | 4.79 |  |  |
| C, SFA-TOC | 84.2 mg C kg^-1^ |  |  |
| Organic matter (105-550°C) | 3.09 % |  |  |
| < 2 µm, % | 2.77 % |  |  |
| Total N | 1070 mg N kg^-1^ |  |  |
| CEC | 19 meq. per 100 g |  |  |
| C:N ratio | 11 |  |  |
