# Supplementary material for: Olivine Weathering in Soil, and Its Effects on Growth and Nutrient Uptake in Ryegrass (Lolium perenne L.): A Pot Experiment
Source: PLoS One. 2012 Aug 9;7(8):e42098. doi: 10.1371/journal.pone.0042098 (PMC3415406; doi:10.1371/journal.pone.0042098)
Supplement: Table S3 — Fertiliser applications. (DOCX) [file pone.0042098.s006.docx]

*Table S3. Fertiliser applications.*

| **Fertiliser product** | **Product dose (g/pot)** | **Corresponds to**  **dose of** |
| --- | --- | --- |
| *starter mixed through soil at filling of pots* | | |
| Calcium ammonium nitrate (CAN) | 1.50 | 80 kg N ha^-1^  6 kg Mg ha^-1^ |
| TripleSuperPhosphate (TSP) | 0.50 | 45 kg P_2_O_5_ ha^-1^  (19.6 kg P ha^-1^) |
| K60 | 0.78 | 80 kg K ha^-1^ |
| Kieserite | 0.17 | 5.6 kg Mg ha^-1^ |
|  |  |  |
| *top dressing, dose per harvest, after each of harvests 1, 2, 3, 4, 5:* | | |
| Calcium ammonium nitrate (CAN) | 1.50 | 80 kg N ha^-1^  6 kg Mg ha^-1^ |
| TripleSuperPhosphate (TSP) | 0.30 | 27 kg P_2_O_5_ ha^-1^  (11.8 kg P ha^-1^) |
|  |  |  |
| *top dressing, dose per harvest, after each of harvests 1, 2, 5* | | |
| K60 | 0.40 | 41 kg K ha^-1^ |
| Potassiumsulphate | 0.48 | 41 kg K ha^-1^ |
|  |  |  |
| *top dressing, dose per harvest, after harvests 3, 4 (avoiding excess sulphur)* | | |
| K60 | 0.78 | 80 kg K ha^-1^ |
