# Supplementary material for: Olivine Weathering in Soil, and Its Effects on Growth and Nutrient Uptake in Ryegrass (Lolium perenne L.): A Pot Experiment
Source: PLoS One. 2012 Aug 9;7(8):e42098. doi: 10.1371/journal.pone.0042098 (PMC3415406; doi:10.1371/journal.pone.0042098)
Supplement: Table S4 — Soil extraction methods; and plant and soil analysis methods. (DOCX) [file pone.0042098.s007.docx]

*Table S4. Soil extraction methods; and plant and soil analysis methods used.*

| Bioavailability: extraction in   - 1. M KH_2_PO_4_ for Si   0.03 M NaCl for Ca  0.01 M CaCl_2_ other elements  1 g soil per 10 ml extractant; shaking end-over-end for two hours | NEN-5704: 1996. Bodem: Monstervoorbehandeling van grond. Extractie met een calciumchloride-oplossing (0.01 mol/L). (Pre-treatment of soil samples. Extraction with calcium chloride solution.)  ISO 14255 Soil quality – Determination of nitrate nitrogen, Ammonium nitrogen and total soluble nitrogen in air-dry soils using calciumchloride solution as extractant. |
| --- | --- |
| P_AL_ phosphorus  Ammonium lactate acetic acid, pH 3.75, at 20+2°C | H. Egner, H. Riehm en W.R. Domingo, Untersuchungen über die chemische Bodenanalyse als Grundlage für die Beurteilung des Nährstoffzustandes der Böden. II Chemische Extraktionsmethoden zur Phosphor- und Kaliumbestimmung, Kungl. Lantbrukshögskolans Ann. 26, 199-215, 1960 (Chemical extraction methods for phosphorus and potassium assessment in soil.)  NEN-EN-ISO-15681-1: 2004. Water quality: Determination of orthophosphate and total phosphorus contents by flow analysis (FIA and CFA). Part 1: Method by flow injection analysis (FIA).  NEN-EN-ISO-15681-2: 2004. Water quality: Determination of orthophosphate and total phosphorus contents by flow analysis (FIA and CFA). Part 2: Method by continuous flow (CFA).  NEN-5973: 2010. Bodem – Bepaling van fosfaat in grond extraheerbaar met een ammoniumlactaat-azijnzuur buffer (P-AL) (Assessment of phosphate extractable in ammonium lactate – acetic acid buffer) |
| 0.43 M HNO_3_  1 g soil per 10 ml extractant; shaking end-over-end 4 hours, centrifuging 10 minutes at 3000 rpm. | V.J.G. Houba, J.J. van der Lee, I. Walinga, I. Novozamsky (1985). Soil analysis, Part 2: Procedures. Department of Soil Science and Plant Nutrition. Wageningen University, The Netherlands. |
| Analysis of plant samples | Analyses by CBLB, Wageningen university.  Plant samples were dried at 70 C.  Destruction for Mg analysis in HNO3-HF-H2O2.  Destruction for Si in HCl-HF  Destruction for all other elements in H2SO4-H2O2-Se  Analysis of N and P by SFA. Analysis of K by F-AES. Other elements by ICP-AES^[[1]](#footnote-1)^ or ICP-MS^[[2]](#footnote-2)^ (depending on value). |
| Analysis of soil samples | Extraction see Table S2.  Analysis by ICP-AES or ICP-MS depending on value; see Table S1. |

1. Inductively Coupled Plasma Atomic Emission Spectroscopy [↑](#footnote-ref-1)
2. Inductively Coupled Plasma Mass Spectrometry [↑](#footnote-ref-2)
