# Supplementary material for: Olivine Weathering in Soil, and Its Effects on Growth and Nutrient Uptake in Ryegrass (Lolium perenne L.): A Pot Experiment
Source: PLoS One. 2012 Aug 9;7(8):e42098. doi: 10.1371/journal.pone.0042098 (PMC3415406; doi:10.1371/journal.pone.0042098)
Supplement: Table S5 — Harvested plant biomass. (DOCX) [file pone.0042098.s008.docx]

*Table S5. Aboveground (harvested) plant biomass^1^, six harvests cumulated.*

| **Treatment** | **Fresh biomass**  (g/pot) | **Dry biomass**  (g/pot) |
| --- | --- | --- |
| Control | 544.0^a^ | 100.2^a^ |
| KIES1 | 540.6^a^ | 96.3^a^ |
| KIES2 | 522.4^a^ | 94.5^a^ |
| OLIV1 | 562.7^a^ | 100.3^a^ |
| OLIV2 | 556.3^a^ | 102.4^a^ |
| OLIV3 | 560.2^a^ | 102.3^a^ |
| OLIV4 | 628.5^b^ | 115.8^b^ |

*^1.^Treatment means sharing the same letter within a column are not significantly different at the 1% level according to a pairwise t-test, while treatment means with no letter in common are significantly different.*
