# Supplementary material for: Olivine Weathering in Soil, and Its Effects on Growth and Nutrient Uptake in Ryegrass (Lolium perenne L.): A Pot Experiment
Source: PLoS One. 2012 Aug 9;7(8):e42098. doi: 10.1371/journal.pone.0042098 (PMC3415406; doi:10.1371/journal.pone.0042098)
Supplement: Table S6 — Total element concentrations in soil (Aqua regia), at last harvest. (DOCX) [file pone.0042098.s009.docx]

*Table S6. Total element concentrations^1^ (Aqua regia) in soil, at the end of the experiment.*

| **Treatment** | **P_total_**  mg kg^-1^ | **Fe_total_**  mg kg^-1^ | **Mg_total_**  mg kg^-1^ | **Ni_total_**  mg kg^-1^ |
| --- | --- | --- | --- | --- |
| Control | 690.0^b^ | 5113^ab^ | 411^a^ | 2.6^a^ |
| KIES1 | 699.5^b^ | 5013^a^ | 454^a^ | 3.1^a^ |
| KIES2 | 704.2^b^ | 5090^ab^ | 441^a^ | 2.9^a^ |
| OLIV1 | 696.8^b^ | 5020^a^ | 592^b^ | 4.7^b^ |
| OLIV2 | 698.5^b^ | 4972^a^ | 1430^c^ | 13.2^c^ |
| OLIV3 | 679.5^b^ | 5442^b^ | 5799^d^ | 60.8^d^ |
| OLIV4 | 605.0^a^ | 8616^c^ | 27761^e^ | 284.2^e^ |

*^1.^Treatment means sharing the same letter within a column are not significantly different at the 1% level according to a pairwise t-test, while treatment means with no letter in common are significantly different.*
