# Supplementary material for: Olivine Weathering in Soil, and Its Effects on Growth and Nutrient Uptake in Ryegrass (Lolium perenne L.): A Pot Experiment
Source: PLoS One. 2012 Aug 9;7(8):e42098. doi: 10.1371/journal.pone.0042098 (PMC3415406; doi:10.1371/journal.pone.0042098)
Supplement: Table S7 — Concentrations of Ca, Mg, Ni, and Si in soil water. (DOCX) [file pone.0042098.s010.docx]

*Table S7. Concentration^1^ of Ca, Mg, Ni, and Si in soil water.*

|  | Feb.17 | | Apr.7 | | |
| --- | --- | --- | --- | --- | --- |
|  | Mg  mg/l | Ni  μg/l | Mg  mg/l | Ni  μg/l | Si  mg/l |
| Control | 4.83^a^ | 5.33^a^ | 2.98^a^ | 2.2^a^ | 0.54^a^ |
| KIES1 |  |  | 3.40^a^ | 3.5^a^ | 1.46^ab^ |
| KIES2 | 7.62^ab^ | 4.70^a^ | 4.14^a^ | 4.2^a^ | 1.00^ab^ |
| OLIV1 |  |  | 4.21^a^ | 3.5^a^ | 0.83^a^ |
| OLIV2 | 9.49^b^ | 21.63^b^ | 5.04^a^ | 6.8^a^ | 0.91^a^ |
| OLIV3 |  |  | 12.92^b^ | 32.8^b^ | 2.44^b^ |
| OLIV4 | 33.13^c^ | 130.58^c^ | 28.02^c^ | 113.7^c^ | 13.30^c^ |

*^1.^Treatment means sharing the same letter within a column are not significantly different at the 1% level according to a pairwise t-test, while treatment means with no letter in common are significantly different.*
